# Metabolic Bariatric Surgery Across the IFSO Chapters: Key Insights on the Baseline Patient Demographics, Procedure Types, and Mortality from the Eighth IFSO Global Registry Report

**DOI:** 10.1007/s11695-024-07196-3

**Published:** 2024-04-09

**Authors:** Wendy A. Brown, Ronald Liem, Salman Al-Sabah, Mehran Anvari, Camilo Boza, Ricardo V. Cohen, Amir Ghaferi, Villy Våge, Jacques Himpens, Lilian Kow, John Morton, Mario Musella, Francois Pattou, Nasser Sakran, Benjamin Clapp, Gerhard Prager, Scott Shikora, on behalf of the IFSO Global Registry Collaboration, Angus Campbell, Jennifer Holland, Jenifer Cottrell, Robin Thompson, Dianne Brown, Wendy Brown, Ian Caterson, Felix Langer, Philipp Beckerhinn, Gehard Prager, Taryel Omerov, Karina Otani, Ricardo Cohen, Karen Barlow, Mehran Anvari, Camilo Boza, Amalia Villaseca, Cunchuan Wang, Wah Yang, Andrea Lazzati, David Lechaux, Valerie Leborgne, Francois Pattou, Mohammad Kermansaravi, Inbal Globus, Nasser Sakran, Mario Musella, Vincenzo Schiavone, Antonio Franzese, Sang Kuon Lee, Salman Al-Sabah, Nik Ritza Kosai Nik Mahmood, Guhan Muthkumaran, Ismail Ahmed Ali, Teh Shunxing, Nursuhadah Mohamed Yusof, José G. Rodríguez Villarreal, Floris Bruinsma, Ronald Liem, Simon Nienhuijs, Jan Willem Greve, Angus Campbell, Jennifer Holland, Jenifer Cottrell, Robin Thompson, Dianne Brown, Andrew MacCormick, Ian Caterson, Hannu Lyyjynen, Villy Vage, Bekkhan Khatsiev, Eugene van Zyl, Tess van der Merwe, Johan Ottoson, Andrew Curry, Benjamin Clapp, John Morton, Aaron J. Bonham, Amanda Stricklen, Rachel Ross, Amir Ghaferi, Nozim Jumaev, Pedro Monsalve, Luis Level

**Affiliations:** 1 Department of Surgery, Australia and New Zealand Bariatric Surgery Registry, Monash University, Level 6, The Alfred Centre, 99 Commercial Road, Melbourne, 3004 Australia; 2 Dutch Audit for the Treatment of Obesity, Heerlen, Netherlands; 3 Department of Surgery, Kuwait University (Kuwait Bariatric Surgery Registry), Kuwait, Kuwait; 4 Ontario Bariatric Registry, Hamilton, ON Canada; 5 Bariatric Surgery Center, Clinica MEDS (Chilean Bariatric Surgery Registry), Santiago, Chile; 6 The Center for the treatment of Obesity and Diabetes – COD Hospital Oswaldo Cruz (Brazilian Registry), Sao Paulo, Brazil; 7 Michigan Bariatric Surgery Collaborative, Ann Arbor, MI USA; 8 Scandinavian Obesity Surgery Registry Norway (SOReg-N), Helse Bergen Health Trust, Bergen, Norway; 9 Delta CHIREC Hospital (Belgian Registry), Brussels, Belgium; 10 Department GI Surgery, Flinders University South Australia (Australian and New Zealand Bariatric Surgery Registry), Adelaide, Australia; 11 Yale School of Medicine (MBSAQIP- Metabolic and Bariatric Surgery Accreditation and Quality Improvement Project), New Haven, CT USA; 12 Advanced Biomedical Sciences Department (Italian Registry), Naples “Federico II” University, Naples, Italy; 13 University of Lille, Integrated Center for Obesity, CHU Lille, Inserm,, Institut Pasteur Lille (SOFFCO-MM Registry), Lille, France; 14 Department of General Surgery, Holy Family Hospital, Nazareth, Israel; 15 The Azrieli Faculty of Medicine Safed, Bar-Ilan University (Israelian Registry), Ramat Gan, Israel; 16 Paul L Foster School of Medicine, Texas Tech University Health Sciences Center El Paso, El Paso, TX 79902 USA; 17 Universitätsklinik Für Allgemeinchirurgie, Vienna, Austria; 18 Department of Surgery, Division of Gastrointestinal and General Surgery, Brigham and Women’s Hospital, Harvard Medical School (MBSAQIP), Boston, MA USA

**Keywords:** Registry, Demographics, Metabolic bariatric surgery, International trends

## Abstract

**Introduction:**

The International Federation for Surgery for Obesity and Metabolic Disorders (IFSO) Global Registry aims to provide descriptive data about the caseload and penetrance of surgery for metabolic disease and obesity in member countries. The data presented in this report represent the key findings of the eighth report of the IFSO Global Registry.

**Methods:**

All existing Metabolic and Bariatric Surgery (MBS) registries known to IFSO were invited to contribute to the eighth report. Aggregated data was provided by each MBS registry to the team at the Australia and New Zealand Bariatric Surgery Registry (ANZBSR) and was securely stored on a Redcap™ database housed at Monash University, Melbourne, Australia. Data was checked for completeness and analyzed by the IFSO Global Registry Committee. Prior to the finalization of the report, all graphs were circulated to contributors and to the global registry committee of IFSO to ensure data accuracy.

**Results:**

Data was received from 24 national and 2 regional registries, providing information on 502,150 procedures. The most performed primary MBS procedure was sleeve gastrectomy, whereas the most performed revisional MBS procedure was Roux-en-Y gastric bypass. Asian countries reported people with lower BMI undergoing MBS along with higher rates of diabetes. Mortality was a rare event.

**Conclusion:**

Registries enable meaningful comparisons between countries on the demographics, characteristics, operation types and approaches, and trends in MBS procedures. Reported outcomes can be seen as flags of potential issues or relationships that could be studied in more detail in specific research studies.

## Introduction

Multiple randomized controlled trials (RCT), cohort studies, and case series from expert centers have demonstrated that metabolic bariatric surgery (MBS) is an effective treatment option for obesity, safely inducing not only weight loss but remission from important obesity-related diseases, including diabetes, hypertension, and cardiovascular disease [1–3]. How these positive results translate in the “real-world” setting remains largely unknown. There is also limited knowledge comparing the uptake and practice of MBS worldwide.

Registries use observational study methods to systematically collect uniform data, which are used to evaluate specified outcomes for a defined population [4]. In the field of MBS, these registries can be used to record the characteristics of the population undergoing MBS, document the types of procedures being performed, capture the safety of surgery through the prospective recording of quality indicators, and track the weight loss, health, and patient-reported outcomes of MBS. There are thirty known national and two complete regional MBS registries, each with an emerging dataset, with some having been shown already to improve outcomes for patients [5].

The International Federation for Surgery for Obesity and Metabolic Disorders (IFSO) has sought to drive collaborations between existing registries so that the positive outcomes achieved by individual registries may be translated globally. IFSO has also sought to help establish registries in other member countries that do not currently have a local registry. The Executive Board of IFSO established the IFSO Global Registry to facilitate these dual goals.

The IFSO Global Registry's mission is *to provide the most credible and transparent information on MBS*. To achieve this mission, the IFSO Global Registry aims to provide descriptive data about caseload and penetrance of surgery for metabolic disease and obesity in member countries and aspire to provide real-world surveillance of procedures and devices.

The first IFSO Global Registry report was produced in 2014. In that report, information was included from 18 countries coming from 5 continents that contributed 100,092 operation records, with 53,197 between the calendar years 2011–2013. The number of operations contributed ranged from one individual center that had entered 24 operation records to over 34,000 each from two countries with established national registries (Sweden and the United Kingdom).

Over time, contributions to the IFSO Global Registry have grown, and by the sixth report, there were 507,298 operations submitted by 50 contributor countries, 10 of whom were national or regional registries. However, including individual-level data from each contributing site created significant challenges for IFSO, particularly with the rigorous standards of data protection required by the General Data Protection Regulations (GDPR).

In 2022, the IFSO Global Registry Committee proposed to the Executive Board of IFSO that future reports include only aggregated data from established national or regional registries using a data dictionary focusing on demographic and descriptive data only. Outcome data was not included as it was inconsistently collected by registries worldwide, making comparisons difficult.

“Aggregated data” means that the data given to the IFSO Global Registry is already analyzed and provided as a mean or a median, meaning individuals cannot be identified. As no individual-level data transfer is required, there is no risk of a GDPR privacy breach. By only including data from national or complete regional registries, selection bias is reduced, making it more likely that the IFSO Global Report accurately represents the activity of an included country or region.

This is a summary of the key findings of the eighth report of the IFSO global registry and the second to contain only aggregated data from established national and regional registries [6].

## Methods

Selected data items were chosen to describe the demographics of people with obesity who undergo MBS, the types of procedures being undertaken, and indicators of perioperative safety. A common data dictionary was developed through a consensus process and included the data items identified as the core for MBS registries through a previous collaboration with Bristol University [7].

All existing MBS registries known to IFSO were invited to contribute to the eighth report. Aggregated data was provided by each MBS registry to the team at the Australia and New Zealand Bariatric Surgery Registry (ANZBSR) and was securely stored on a Redcap™ database housed at Monash University, Melbourne, Australia.

**Fig. 1:**
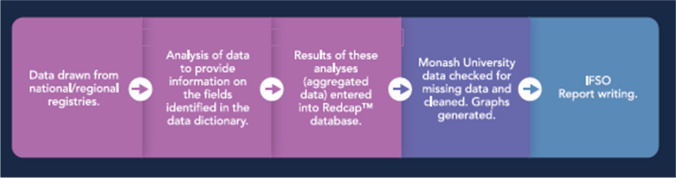
Process for data collection and collation

Data was checked for completeness by the ANZBSR team and was then analyzed by the IFSO Global Registry Committee. Prior to the finalization of the report, all graphs were circulated to contributors and to the global registry committee of IFSO to ensure data accuracy (Fig. 1).

### Statistical Methods

Aggregated data from each country or region is being compared, meaning that statistical comparisons are impossible as it is not possible to compare data that has already been analyzed and described as medians or means.

### Caveats

Given that all contributing registries are well established and already collect data according to their definitions, it was not possible to completely align the data-set against the common data dictionary.

Not all countries or regions collected all of the data items that were chosen for the global report. Where an item is not collected, the country or region is not included in the reported information.

## Results

### Participants

**Fig. 2:**
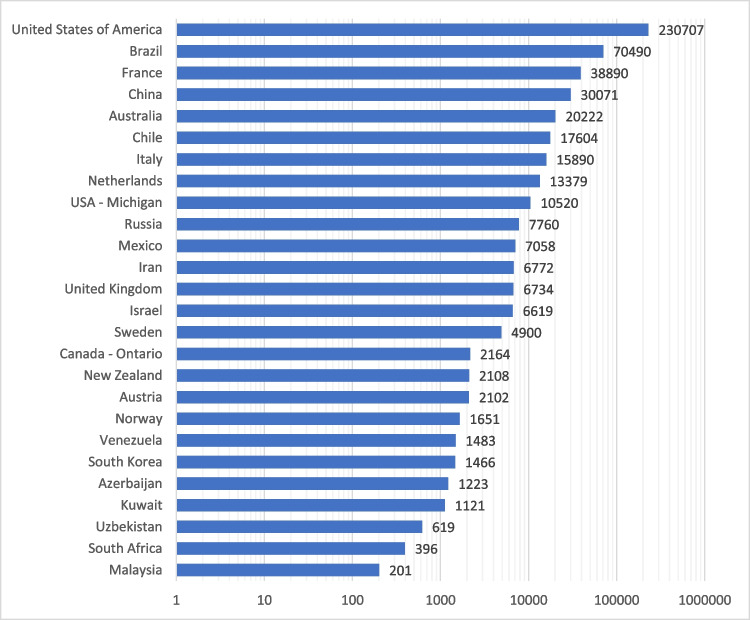
Number of metabolic bariatric surgical procedures per country or region. NB: X-axis is a logscale. Twenty-four countries and 2 regional registries contributed 502,150 procedures, with 449,815 (89.5%) primary procedures and 52,335 (10.5%) revisional procedures. Michigan is a state in the United States of America, and 39 of its 41 sites also contribute to the MBSAQIP (USA) Registry, meaning 10,437 procedures are potentially represented twice in this graph. The UK data is from 2021

Data were contributed by 24 countries and 2 complete regional registries (81.3% of all known registries), including information on 502,150 completed MBS in either 2021 (United Kingdom) or 2022 (rest of the world) (Fig. 2).

Most procedures were primary MBS procedures (*n* = 449,815 (89.6%)), meaning they were the first MBS procedure a participant had undertaken. Revisional procedures (*n* = 52,335 (10.4%)) included those procedures undertaken to convert one MBS procedure to another or to correct a side effect of the procedure. The highest rates of revisional MBS were seen in Australia (Table 1).

**Table 1:** Number of primary and revisional procedures by country or region

Country or region	Primary (*n*)	Revisional (*n*)	Primary %	Revisional %
United States of America	204,324	52,335	88.6%	11.4%
Brazil	63,442	7,048	90.0%	10.0%
France	32,490	6,400	83.5%	16.5%
China	29,823	248	99.2%	0.8%
Chile	16,855	749	95.7%	4.3%
Australia	16,308	3,914	80.6%	19.4%
Italy	14,391	1,499	90.6%	9.4%
Netherlands	12,327	1,052	92.1%	7.9%
USA—Michigan	9,319	1,201	88.6%	11.4%
Russia	7,345	415	94.7%	5.3%
Mexico	6,649	409	94.2%	5.8%
Iran	6,631	141	97.9%	2.1%
United Kingdom	6,118	616	90.9%	9.1%
Israel	5,556	1,063	83.9%	16.1%
Sweden	4,677	223	95.4%	4.6%
Canada—Ontario	2,064	100	95.4%	4.6%
New Zealand	2,014	94	95.5%	4.5%
Austria	1,817	285	86.4%	13.6%
Norway	1,575	76	95.4%	4.6%
South Korea	1,406	60	95.9%	4.1%
Venezuela	1,351	132	91.1%	8.9%
Azerbaijan	1,191	32	97.4%	2.6%
Kuwait	934	187	83.3%	16.7%
Uzbekistan	614	5	99.2%	0.8%
South Africa	394	2	99.5%	0.5%
Malaysia	200	1	99.5%	0.5%

### Data Completeness

Sweden, Michigan (USA), Netherlands, Ontario (Canada), France, and Israel reported complete, or near-complete, case ascertainment—meaning their local Registry captured every person who underwent a metabolic bariatric procedure. MBSAQIP (USA) reported 82.4%, Norway 89%, and Australia 82.2% case ascertainment. Other countries did not report case ascertainment.

### Demographics and Setting

**Fig. 3:**
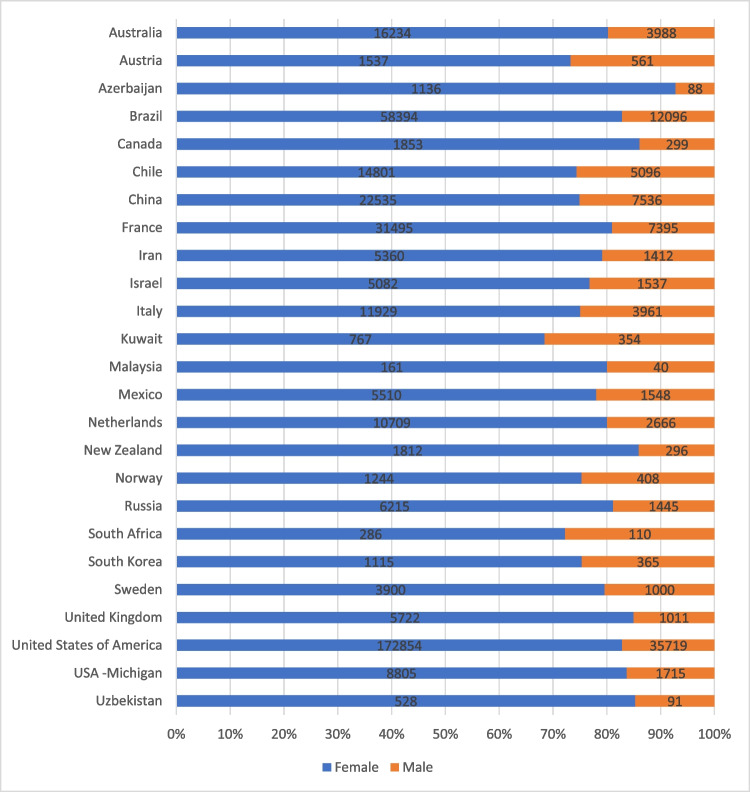
Proportion of participants in registries recorded as female or male. Differences in overall numbers in each registry recording sex may reflect people identifying as a sex other than male or female or the field being incompletely recorded

Twenty-five registries provided data on sex, being recorded as male or female. The majority of participants were female (81.1%). Females predominated in all contributing registries (Fig. 3).

Twenty-one registries provided the median age on the day of surgery. The median age day of surgery ranged from 31 years (IQR 25–37 years) in China to 44.7 years (IQR 35.8–53.5 years) in the United Kingdom (Table 2). Participants undergoing primary MBS were younger than those undergoing revisional MBS (Table 3). Males tended to be older than females, apart from in China, Kuwait, South Korea, Iran, Italy, and Malaysia, where they were younger (Table 3).

**Table 2:** Median age on the day of surgery by country or region. Age day of surgery for all procedures by country or region

Country	Median	Lower IQR	Upper IQR
Australia	42.4	33.9	51.5
Austria	41	39.8	48
Canada—Ontario	43.8	36.1	51.8
China	31	25	37
France	41	32	51.1
Iran	38	31	45
Israel	37.2	28.2	47.2
Italy	45	35	52
Kuwait	34	25	42
Malaysia	41	35.5	47.5
Netherlands	45	34	54
New Zealand	43.6	35.2	51.9
Norway	42	32.5	51.2
Russia	40.8	34.4	48.8
South Africa	43	37	50
South Korea	35	29	42
Sweden	41	32	50
United Kingdom	44.7	35.8	53.5
United States of America	43	35	52
USA- Michigan	43	35	52
Uzbekistan	41	34	45

**Table 3:** Median age on the day of surgery by country or region. Age day of surgery by sex for primary and revisional procedures by country or region

	Primary metabolic bariatric procedures									Revisional Metabolic Bariatric Procedures								
	Female			Male			All			Female			Male			All		
	Median	Lower IQR	Upper IQR	Median	Lower IQR	Upper IQR	Median	Lower IQR	Upper IQR	Median	Lower IQR	Upper IQR	Median	Lower IQR	Upper IQR	Median	Lower IQR	Upper IQR
Australia	39.9	32.2	49.4	42.6	34.8	51	40.5	32.7	49.8	49.5	41	57	51.2	42.6	58.6	49.9	41.2	57.4
Austria	39	40.4	48	43.2	41	50.5	40	40.7	48.7	48	27.5	40.9	54	29.4	44.6	49	27.6	41.7
Canada—Ontario	43.2	35.6	51.4	46.7	39.5	54.1	43.7	37.2	53.9	44.8	37.7	51.3	48.3	40.4	52.9	45.5	37.8	51.8
China	31	25	37	30	23	36	30	24.4	37	43.7	32.8	49.4	45.3	34.6	48.9	44.7	34.7	46.9
Iran	38	31	46	36	30	42	38	31	45	40	34	48	39	35	48	40	34	48
Israel	34.9	26.7	44.6	36.9	27.4	46.8	35.3	26.8	45.2	46.2	37.18	54.2	48.7	38.2	55.3	46.8	37.4	54.4
Italy	44	34	52	36	45	52	44	34	52	45	32	57	48	39	57	45	32	57
Kuwait	33	24	41	31	22	39	32	23	42	40	32	47	42	34	48	41	32	47
Malaysia	41	36	49	40	35	45.8	41	36	49	49	0	0	0	0	0	49	0	0
Netherlands	43	33	52	48	37	55	44	34	53	50	41	56	52	47	59	50	42	57
New Zealand	42.7	34.7	51.5	46.3	37.4	53.7	43.3	35.1	51.8	47.5	39.6	56.2	51.4	44.3	58.3	48.2	40.1	57
Norway	40.7	32	50.3	45.5	35	52.5	41.9	32.4	51	46.3	35.2	54.2	45.9	36.4	56.6	46.3	35.8	55.4
Russia	40.5	25.3	48.6	41.4	34.6	49.2	40.6	34.2	48.6	44.7	39.5	51.7	46	39.7	52.9	44.9	39.6	52
South Africa	41	36	49	46	38	54	43	37	50	50	50	50	48	48	48	49	48	50
South Korea	36	29	43	33	29	40	35	29	42	38.5	34	43.3	41	30.8	48	39	33.8	43.3
Sweden	40	32	49	44	35	52	40	34	50	46	37	54	48	39.5	52	46	37.5	54
United Kingdom	43.3	34.7	52.5	47.6	38.5	55.4	43.9	35.2	53	49.7	42.2	56.5	52.3	47.3	58.8	50.3	42.6	56.9
United States of America	42	34	51	45	37	54	43	34	52	49	42	57	52	44	59	50	42	57
USA-Michigan	42	34	51	45	38	54	42	34	51	48	40	55	52	43.5	61	48	41	56
Uzbekistan	36	28	43	44	30	49	40	29	46	38	36	40	46	44	48	42	40	44

The median BMI on the day of a primary MBS was collected by 21 registries and ranged from 36.1 kg/m^2^ for women in China to 47.7 kg/m^2^ for males in South Africa (Table 4).

**Table 4:** BMI on the day of primary MBS by country and sex

	Female			Male			All		
	Median	Lower IQR	Upper IQR	Median	Lower IQR	Upper IQR	Median	Lower IQR	Upper IQR
China	36.1	32.1	41	40.4	35.9	46.1	37.4	33.1	42.9
South Korea	37.3	34.3	41.2	41.0	36.5	46.4	38.1	35.0	42.6
Sweden	40.2	36.7	44.3	42.3	38.7	46.7	40.6	37.2	44.8
Israel	41	38.5	44	42	39.1	45.7	41	38.7	44.5
Norway	40.6	37.4	44.6	42.9	39.3	47.5	41.1	37.7	45.2
Netherlands	41.6	39.3	45	41.9	39	45.7	41.7	39.2	45.1
Malaysia	43.5	35.3	45.9	44	38.2	50.5	42	36	46
Kuwait	41.5	38.8	46.1	43.1	40.1	49.8	42	39.2	47.1
Iran	41	38	45	43	40	47	42	39	45
Italy	41	38	45	43	39	48	42	38	46
Australia	41.8	37.7	47.1	43.3	39.2	48.9	42.1	38	47.5
Russia	41.5	37.1	47.3	45.3	41.0	51.1	42.2	37.4	47.8
Uzbekistan	42	38	44	44	40	46	43	39	45
New Zealand	43.0	38.9	48.5	44.6	40.2	51.6	43.3	39.1	48.8
United States of America	43	40	49	45	40	51	44	40	50
Austria	43.7	40.4	48	45.1	41	50.5	44	40.7	48.7
USA-Michigan	44.1	40.3	49.3	45.8	41.2	51.6	44.3	40.4	49.7
United Kingdom	45	40.6	50.4	46.5	41.5	52.3	45.1	40.8	50.7
Canada—Ontario	45.5	41.8	51.2	47.5	42.4	53.2	45.6	41.8	51.4
South Africa	45.2	41.3	52.2	47.7	42	55.2	45.8	41.6	53

*France collects BMI information differently from other registries. Their data is included for completeness below:

### Procedure Types

**Fig. 4:**
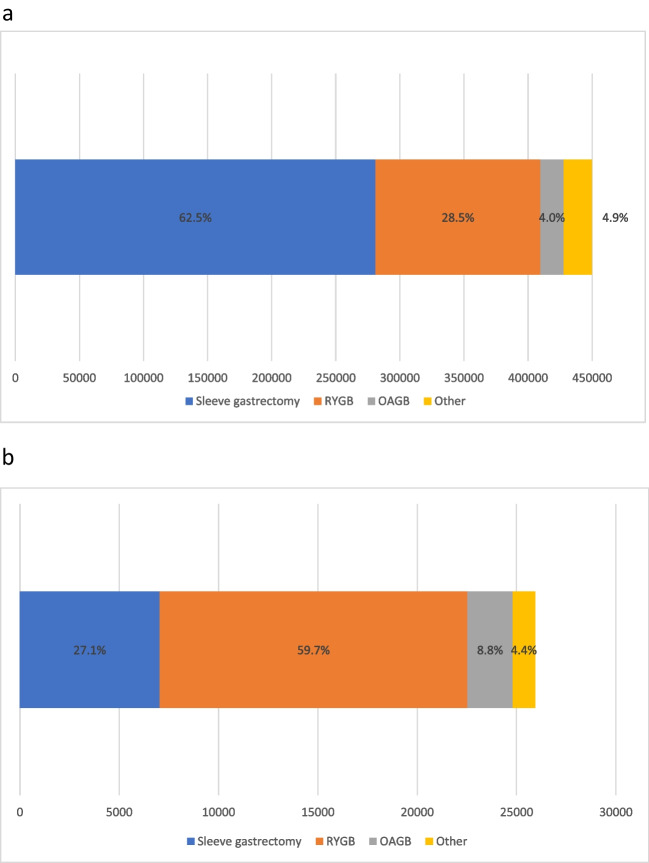
MBS procedure type. *Potential for procedures to be represented twice due to possible overlaps with the datasets of the USA and Michigan. **a** Primary MBS procedures (*n* = 449,815). **b** Revisional procedures (*n* = 31,278; excluded 21,057 cases labelled revision/conversion cases from United States of America that did not have a procedure type specified)

The most commonly performed primary procedure worldwide was sleeve gastrectomy (SG) (Fig. 4a), and the most commonly performed revisional procedure was Roux-en-Y gastric bypass (RYGB) (Fig. 4b) with the caveat that the MSBAQIP (USA) has an additional category being “revisional/conversion” surgery that does not specify the subsequent procedure type.

**Fig. 5:**
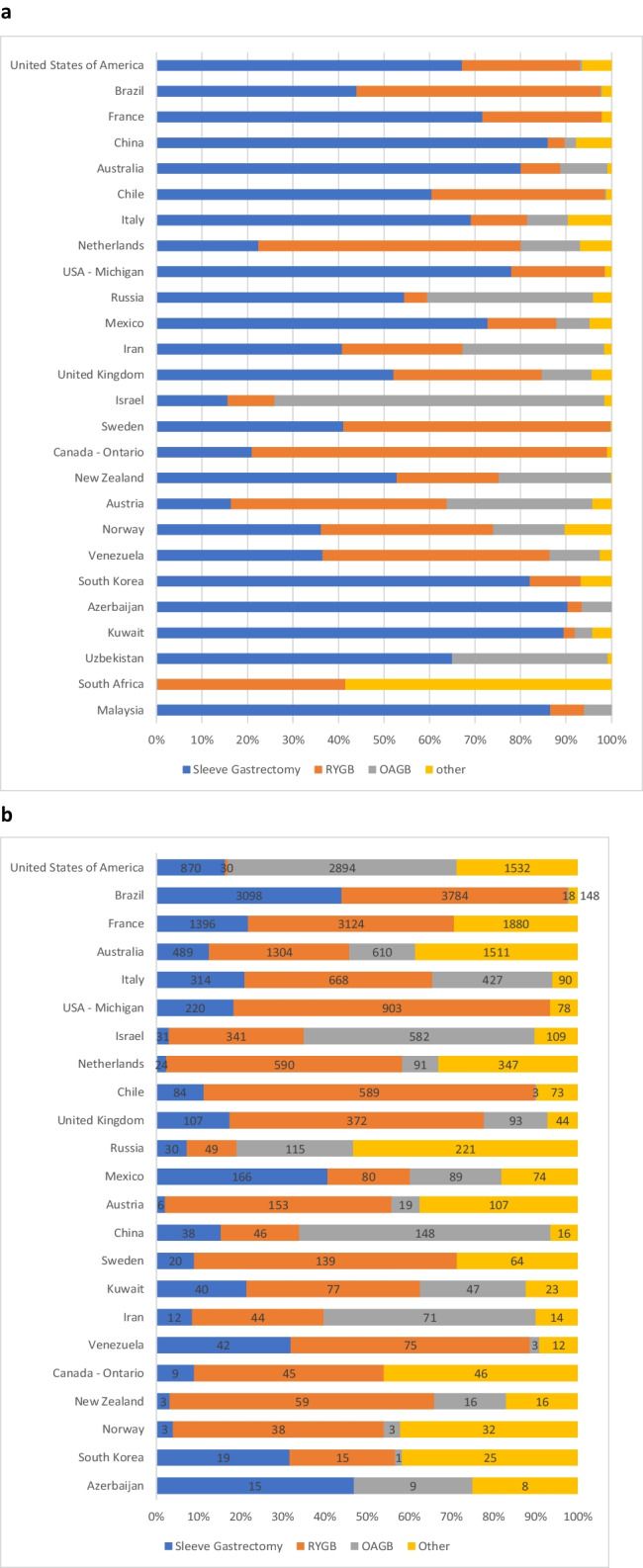
**a** Primary MBS types by country or region (*n* = 449,815). **b** Revisional MBS types by country or region (*n* = 52,335). Malaysia (*n* = 1), Uzbekistan (*n* = 5), and South Africa (*n* = 3) cannot be graphically displayed. The United States of America reported an additional 21,057 revisional cases labelled “revision/conversion” that are not able to be displayed graphically. This means the breakdown of procedures displayed in this graph may not be representative

However, there are differences between countries when considering primary MBS. While the United States of America (USA) reported 140,339 primary SG (68.8%), RYGB was the most commonly reported primary MBS in Brazil, Venezuela, Netherlands, Norway, Ontario (Canada), Austria, and Sweden, with one-anastomosis gastric bypass (OAGB) being the most common procedure in Israel and “other” procedures predominating in South Africa (Fig. 5a).

Most procedures were completed laparoscopically, with the proportion of robotic cases being higher in the revisional setting (Table 5). The two USA-based registries reported the overall highest use of robotic systems.

**Table 5:** Operative approach by country or region

	Primary MBS							Revisional MBS						
	Laparoscopic	Open	Endoscopic	Robotic	Unspecified	Laparoscopic rate	Robotic rate	Laparoscopic	Open	Endoscopic	Robotic	Unspecified	Laparoscopic rate	Robotic rate
Australia	16,151	2	1	117	37	99.0%	0.7%	3769	21	78	35	11	96.3%	0.9%
Austria	1738	4	1	42	32	95.7%	2.3%	271	10	0	1	3	95.1%	0.4%
Canada—Ontario	2050	13	0	0	1	99.3%	0.0%	98	2	0	0	0	98.0%	0.0%
France	32,408	82	0	0	0	99.7%	0.0%	6,261	139	0	0	0	97.8%	0.0%
Iran	6624	7	0	0	0	99.9%	0.0%	141	0	0	0	0	100.0%	0.0%
Israel	5548	6	0	0	2	99.9%	0.0%	1057	6	0	0	0	99.4%	0.0%
Italy	12,926	5	0	88	1372	89.8%	0.6%	884	14	4	2	595	59.0%	0.1%
Kuwait	900	0	32	2	0	96.4%	0.2%	178	2	0	7	0	95.2%	3.7%
Mexico	6526	13	112	11	1	97.9%	0.2%	375	0	20	0	0	94.9%	0.0%
Netherlands	12,306	9	12	0	0	99.8%	0.0%	1039	10	3	0	0	98.8%	0.0%
New Zealand	2014	0	0	0	0	100.0%	0.0%	93	0	0	0	1	98.9%	0.0%
Norway	1576	0	0	0	0	100.0%	0.0%	74	2	0	0	0	97.4%	0.0%
Russia	7249	73	3	0	0	99.0%	0.0%	294	18	1	0	0	93.9%	0.0%
South Africa	393	1	0	0	0	99.7%	0.0%							
Sweden	4665	7	0	0	5	99.7%	0.0%	218	4	0	0	1	97.8%	0.0%
United Kingdom	6012	13	18	75	0	98.3%	1.2%	597	7	2	9	1	96.9%	1.5%
USA—Michigan	6369	3	0	2,947	0	68.3%	31.6%	828	17	0	356	0	68.9%	29.6%
United States of America	203,060	90	1174	61,425	0	76.4%	23.1%	4152	132	1,042	991	0	65.7%	15.7%
Uzbekistan	614	0	0	0	0	100.0%	0%	5	0	0	0	0	100%	0%
Venezuela	1484	0	0	0	0	100.0%	0%							

### Diseases Associated with Obesity—Diabetes

The definition of diabetes varied between registries (Table 6) and included information on the number of people undergoing MBS and concurrent diabetes from 22 registries. The proportion of people undergoing MBS who also have diabetes ranges from 47.4% for Azerbaijan to 7.7% for France and 11% for the Norwegian and Australian Registries. Men are overrepresented in the proportion of people with diabetes undergoing MBS (Table 7).

**Table 6:** Definition of diabetes by registry

Country	Definition used
Australia	Diabetes Status at the Baseline is determined by the patient identifying themselves as having diabetes at the operation and having treatment for their diabetes
Canada—Ontario	Diabetes status at baseline is determined by the patient’s primary care physician at baseline
China	T2DM was defined as fasting blood glucose > 7.0 mmol/L, or/and random blood glucose > 11.1 mmol/L, or/and 2-h blood glucose after a 75-g oral glucose tolerance test > 11.1 mmol/L, or/and the use of antihyperglycemic drugs
Iran	Diabetes status at baseline is determined by the history of the previous diabetes diagnosis, receiving diabetes treatment or diagnosis based on lab tests (FBS > 126 or HbA1C > 6.5) at the time of operation
Israel	Self-reported by the patient before surgery
Italy	Diabetes status is determined according to ADA (American Diabetes Association) Diabetes Care 2014; 37(S 1): S81-90
Kuwait	Patients with type 2 diabetes
Netherlands	> 42 mmol HbA1c/ mol HbA
New Zealand	Diabetes Status at the Baseline is determined by the patient identifying themselves as having diabetes at the operation and having treatment for their diabetes
Norway	Treated with medication
Russia	Diabetes status at baseline is determined by the patient self-reporting as having diabetes at the operation or having diabetes treatment
South Africa	ADA criteria for DM/pre-diabetes and gestational DM
South Korea	Diabetes status at baseline is determined by the patient identifying themselves as having diabetes at the time of the operation and having diabetes treatment
Sweden	Patients with type 2 diabetes and with medication f
United Kingdom	Patients with type 2 diabetes at surgery who are treated with diabetes medication
USA—Michigan	Type I or Type II diabetes: (Disease marked by high levels of sugar in the blood that occurs because the body does not respond correctly to insulin, a hormone released by the pancreas) non-insulin-dependent diabetes mellitus (NIDDM), adult-onset diabetes mellitus treated with (please check all that apply): Diet, Oral Medications, Insulin-dependent, Non-Insulin Injectables
Uzbekistan	Established type 2 diabetes mellitus before surgery, who are treated with diabetes medications

**Table 7:** Proportion of people undergoing primary MBS who also have diabetes

Country or region	Female			Male			All		
	Diabetes (*n*)	Total (*n*)	% with diabetes	Diabetes (*n*)	Total (*n*)	% with diabetes	Diabetes (*n*)	Total (*n*)	% with diabetes
France	NA	NA	6.5%	NA	NA	13.1%	NA	NA	7.7%
Norway	96	1180	8.1%	77	396	19.4%	173	1576	11.0%
Australia	1213	12,341	9.8%	513	3149	16.3%	1726	15,490	11.1%
Iran	597	5245	11.4%	174	1386	12.6%	771	6631	11.6%
Netherlands	988	9815	10.1%	502	2485	20.2%	1490	12,302	12.1%
Sweden	394	3665	10.8%	177	962	18.4%	571	4627	12.3%
Russia	643	5944	10.8%	273	1384	19.7%	916	7328	12.5%
New Zealand	205	1727	11.9%	63	279	22.6%	268	1995	13.4%
Israel	520	4247	12.2%	236	1309	18.0%	756	5556	13.6%
Kuwait	82	602	13.6%	52	305	17.0%	134	907	14.8%
Malaysia	24	160	15.0%	7	40	17.5%	31	200	15.5%
United Kingdom	690	5054	13.7%	261	914	28.6%	951	5969	15.9%
Canada—Ontario	260	1764	14.7%	69	284	24.3%	329	2048	16.1%
Austria	67	399	16.8%	57	150	38.0%	124	549	22.6%
Italy	1700	9236	18.4%	1400	3094	45.2%	3100	12,330	25.1%
USA-Michigan	2091	7711	76.5%	641	1608	23.5%	2732	9319	29.3%
United States of America	36,896	131,228	28.1%	11,022	25,101	43.9%	47,918	156,329	30.7%
South Africa	66	258	25.6%	47	93	50.5%	113	351	32.2%
Uzbekistan	166	525	31.6%	49	89	55.1%	215	614	35.0%
South Korea	315	947	33.3%	141	329	42.9%	456	1303	35.0%
China	NA	NA	NA	NA	NA	NA	11,571	29,823	38.8%
Azerbaijan	507	1104	45.9%	57	87	65.5%	564	1191	47.4%

*NA, not available

### Mortality Following MBS

The mortality rate following MBS is low in all 19 registries that report this variable. Mortality rates are lower for primary than revisional procedures (Table 8).

**Table 8:** Mortality following MBS

Country or region	Primary				Revisional			
	Deaths (*n*)	Total* (*n*)	Mortality rate	Known cases	Deaths (*n*)	Total* (*n*)	Mortality rate	Known cases
Australia	6	15,044	0.04%	92.2%	2	3703	0.05%	94.6%
Austria	0	1023	0.00%	56.3%	0	197	0.00%	69.4%
Canada—Ontario	0	2064	0.00%	100.0%	0	100	0.00%	100.0%
China	3	NS	NS	NS	3	NS	NS	NS
France	22	32,490	0.10%	100.0%	16	6400	0.30%	100.0%
Iran	9	6631	0.14%	100.0%	2	141	1.42%	100.0%
Israel	1	5556	0.02%	100.0%	2	1063	0.19%	100.0%
Italy	2	14,391	0.01%	100.0%	3	1499	0.20%	100.0%
Malaysia	0	200	0.00%	100.0%	0	1	0.00%	100.0%
Netherlands	4	12,327	0.03%	100.0%	3	1052	0.29%	100.0%
New Zealand	0	1881	0.00%	93.4%	0	88	0.00%	93.6%
Norway	0	1576	0.00%	100.0%	0	76	0.00%	100.0%
Russia	2	7345	0.03%	100.0%	1	315	0.32%	100.0%
South Africa	1	394	0.25%	100.0%	0	2	0.00%	100.0%
South Korea	0	1303	0.00%	91.8%	0	38	0.00%	63.3%
Sweden	0	4677	0.00%	100.0%	0	223	0.00%	100.0%
United Kingdom	2	2747	0.07%	44.9%	1	224	0.45%	36.4%
United States of America	149	204,175	0.07%	99.9%	59	5267	1.12%	98.9%
USA Michigan	2	9319	0.02%	100.0%	3	1201	0.25%	100.0%

*total* number of procedures with known death status

*Mortality rate* percentage of patients readmitted out of all procedures where death status is known

*Known cases* percentage of procedures where death status is known out of the total number of procedures. Excludes unknown/missing values

*NS* not stated

## Discussion

These data are from the eighth report of the IFSO Global Registry [6]. All IFSO chapters are represented in this report, and 81.3% of known registries have included their data. As the data provided by each registry is already analyzed (aggregated), it is impossible to compare data between contributors statistically; however, trends are easily seen.

More women than men seek MBS in every contributing registry. In most countries, women undergoing MBS are younger than their male counterparts, with the exception of China, Kuwait, South Korea, Iran, Italy, and Malaysia. Women are also more likely to have a lower BMI than men.

While women are more likely to undergo MBS than men, men who undergo MBS are more likely to have diabetes. These data may suggest that the main driver for men seeking MBS is health concerns rather than weight loss alone. These sex-based differences are important considerations when designing patient-focused educational material and guidelines for MBS.

China (37.5 kg/m^2^) and South Korea (38.1 kg/m^2^) reported the lowest median BMI on the day of primary MBS. These countries also reported high rates of diabetes in their participants undergoing primary MBS, at 38% and 35%, respectively. Other countries that reported high rates of diabetes in their participants also reported higher BMI on the day of surgery (South Africa, Uzbekistan, USA, Michigan, and Ontario). This difference most likely reflects the increased risk of metabolic diseases in Asian populations at lower BMI [8].

Similar to previous IFSO Surveys [9], SG is the most popular primary MBS globally; however, these data are possibly skewed by the high proportion of primary participants from the USA. Of note, there are nine registries where RYGB, OAGB, or other procedures are reported more often as MBS primary procedures than SG. This is a change from previous reports [9] and is a trend away from SG as the preferred primary procedure that should be monitored.

Revisional surgery rates were the highest in Australia, France, and Israel. This may reflect the higher proportion of primary gastric bands and SG previously performed in these countries [9]. As the rates of primary gastric band procedures continue to fall globally, it will be interesting to see if the need for revisional surgery changes in these countries It will also be important to monitor if the rate of revisional is impacted by the introduction of effective pharmacotherapies [10].

Mortality following MBS was a rare event in all registries that measured this important metric, with rates ranging from 0 to 0.25% in the primary setting and 0–1.42% in the revisional setting, highlighting the safety of MBS.

The strength of this study was the number of included registries, the use of aggregated data that had already been cleaned and checked by contributing registries, and the representation of all IFSO Chapters. The limitations include the lack of clarity about case acquisition rates from most registries, inconsistencies in data definition, and missing data items from some registries.

## Conclusions

This report highlights the opportunities that registries offer to make meaningful comparisons between countries on the demographics, characteristics, operation types and approaches, and trends in MBS procedure types. Reported outcomes can be seen as flags of potential issues or relationships that could be studied in more detail in specific research studies.
